# Specialization in Medicine

**Published:** 1896-11

**Authors:** 


### SPECIALIZATION IN MEDICINE.

Dr. S. W. Kelly, Cleveland, O., recently read a paper on Pediatrics,—Past, Present, and Prospective, in which he gives the results of certain inquiries which he has made by letter in this country and in Britain concerning the advisability of teaching children's diseases separately from general medicine and surgery, and of practicing "Pediatrics" as a specialty. The answers he received were, as was of course to be expected, divided, and somewhat evenly. Some gentlemen, especially Mr. Owen, London, were thoroughly against specializing this branch of practice, one seeing "no more reason for isolating the teaching of pediatrics from general medicine and general surgery than there would be in separating senile diseases in the same way." Mr. Owen wrote, "A thousand times *No.* I should think it the worst thing in the world for a man to give up his time to the study of *children's diseases.* At Great Ormond Street Hospital, the Mother of Children's Hospitals in England, we will not have a man on the staff unless he is, or is likely to be shortly, on the staff of a general hospital."

It seems to us that there is some difference between teaching and practicing children's diseases. Wards and hospitals are most wisely set aside for children, and the teaching material there should not be neglected; besides, there is no doubt that in a course of lectures and demonstrations confined to diseases in young people certain lights are thrown upon the subject which, though they might be taught along with general medicine, are, as a matter of fact not there so thoroughly taught. We are firm believers in the wisdom of senior students and postgraduates taking special courses in diseases of children, but the advisability of separating them altogether in practice wants decided qualification. We cannot see, however, what objection there is to a physician who has proved himself specially adapted to work among children making them the chief object of his attention so long as he does not cut himself off from the consideration of disease as it affects mankind generally. This is true to a greater or less extent of all specialties, and there is no doubt that the man who starts his career as a specialist in any branch of medicine whatsoever, without having worked for at least some five years among general diseases, handicaps himself as well as his patients.

